# Proton Pump Inhibitors and Cyclin-Dependent Kinase 4/6 Inhibitors in Patients With Breast Cancer

**DOI:** 10.1093/oncolo/oyae015

**Published:** 2024-02-10

**Authors:** Kaori Takahashi, Ryuji Uozumi, Toru Mukohara, Tetsu Hayashida, Midori Iwabe, Hirotoshi Iihara, Kanako Kusuhara-Mamishin, Yuko Kitagawa, Masami Tsuchiya, Mika Kitahora, Aiko Nagayama, Shinkichi Kosaka, Yoshimi Asano-Niwa, Tomoko Seki, Koji Ohnuki, Akio Suzuki, Fumiko Ono, Manabu Futamura, Hitoshi Kawazoe, Tomonori Nakamura

**Affiliations:** Division of Pharmaceutical Care Sciences, Center for Social Pharmacy and Pharmaceutical Care Sciences, Keio University Faculty of Pharmacy, Tokyo, Japan; Department of Industrial Engineering and Economics, Tokyo Institute of Technology, Tokyo, Japan; Department of Medical Oncology, National Cancer Center Hospital East, Chiba, Japan; Department of Surgery, Keio University School of Medicine, Tokyo, Japan; Department of Pharmacy, Miyagi Cancer Center, Miyagi, Japan; Department of Pharmacy, Gifu University Hospital, Gifu, Japan; Department of Pharmacy, National Cancer Center Hospital East, Chiba, Japan; Department of Surgery, Keio University School of Medicine, Tokyo, Japan; Department of Pharmacy, Miyagi Cancer Center, Miyagi, Japan; Department of Pharmacy, Gifu University Hospital, Gifu, Japan; Department of Surgery, Keio University School of Medicine, Tokyo, Japan; Department of Surgery, National Hospital Organization Mito Medical Center, Ibaraki, Japan; Department of Breast Surgery, Gifu University Hospital, Gifu, Japan; Department of Surgery, Keio University School of Medicine, Tokyo, Japan; Department of Breast Surgery, Miyagi Cancer Center, Miyagi, Japan; Department of Pharmacy, Gifu University Hospital, Gifu, Japan; Department of Surgery, Keio University School of Medicine, Tokyo, Japan; Department of Breast Surgery, Gifu University Hospital, Gifu, Japan; Division of Pharmaceutical Care Sciences, Center for Social Pharmacy and Pharmaceutical Care Sciences, Keio University Faculty of Pharmacy, Tokyo, Japan; Division of Pharmaceutical Care Sciences, Keio University Graduate School of Pharmaceutical Sciences, Tokyo, Japan; Division of Pharmaceutical Care Sciences, Center for Social Pharmacy and Pharmaceutical Care Sciences, Keio University Faculty of Pharmacy, Tokyo, Japan; Division of Pharmaceutical Care Sciences, Keio University Graduate School of Pharmaceutical Sciences, Tokyo, Japan

**Keywords:** breast cancer, palbociclib, abemaciclib, proton pump inhibitor, propensity score

## Abstract

**Background:**

Proton pump inhibitors (PPIs) reduce the bioavailability of several anticancer drugs. The impact of PPIs co-administered with cyclin-dependent kinase 4 and 6 inhibitors is controversial. We aimed to clarify whether the concomitant use of PPIs impacts palbociclib and abemaciclib effectiveness in breast cancer treatment.

**Patients and Methods:**

This multicenter, retrospective, observational study, conducted across 4 medical institutions in Japan, consecutively included patients with endocrine-resistant metastatic breast cancer, receiving palbociclib or abemaciclib between December 2017 and August 2022. Propensity score-matched analyses were performed. Treatment efficacy and safety with and without PPIs were compared. Progression-free survival and overall survival were estimated using the Kaplan-Meier method and compared using a log-rank test. A Cox proportional hazards model was used to estimate the hazard ratio.

**Results:**

The study included 240 patients. After 1:1 matching, 112 patients were treated with and without PPIs. The median progression-free survival period was 1.2 years in the PPI group and 1.3 years in the non-PPI group (hazard ratio, 1.19; 95% CI, 0.70-2.02). The median overall survival period was 3.6 years in the PPI group, whereas it was not reached in the non-PPI group (hazard ratio, 1.23; 95% CI, 0.61-2.47). Consistent results were obtained for subgroups receiving palbociclib (*n* = 177) and abemaciclib (*n* = 63) without propensity score matching. Adverse event incidence and severity were similar in both groups.

**Conclusion:**

The effectiveness of cyclin-dependent kinase 4/6 inhibitors is unlikely to be affected by concomitant PPI use. Future prospective pharmacokinetic studies are warranted.

## Teaser

The impact of the co-administration of proton pump inhibitors with cyclin-dependent kinase 4 and 6 inhibitors remains controversial. This study clarified whether the concomitant use of proton pump inhibitors impacts the effectiveness of palbociclib and abemaciclib in patients with breast cancer.

**Implications for Practice**

The impact of the co-administration of proton pump inhibitors (PPIs) with cyclin-dependent kinase 4 and 6 inhibitors is controversial. This study clarified whether the concomitant use of PPIs impacts the effectiveness of palbociclib and abemaciclib in patients with endocrine-resistant metastatic breast cancer using real-world data. Importantly, we identified that progression-free survival and overall survival were unaffected by the concomitant use of PPIs. The findings of this study suggest that further consideration should be given to changing the prescription of PPIs for patients receiving palbociclib or abemaciclib.

## Introduction

Breast cancer (BC), the most common cancer and the fourth leading cause of cancer-related death in women, affected approximately 2.3 million individuals worldwide in 2020.^1^ Japan reported approximately 97,000 cases of BC in 2019.^2^ Approximately one in 9 Japanese women are estimated to develop BC. Unfortunately, all patients with metastatic BC (mBC) are incurable. Approximately 60% of mBC cases are luminal, hormone receptor-positive, and human epidermal growth factor receptor type 2 (HER2) negative.^3^

Compared with traditional endocrine monotherapy, combination therapy with cyclin-dependent kinase 4 and 6 (CDK4/6) inhibitors, consisting of palbociclib, ribociclib, and abemaciclib, and endocrine therapy, such as aromatase inhibitors or antiestrogen, has remarkably prolonged progression-free survival (PFS) in patients with hormone receptor-positive and HER2-negative mBC.^4-9^ The use of CDK4/6 inhibitors plus endocrine therapy is recommended as the new standard of care for first- or second-line treatment of pre- and postmenopausal patients with hormone receptor-positive and HER2-negative mBC.^10,11^ Notably, CDK4/6 inhibitors have a different mechanism of action than other cancer chemotherapies. They prevent cellular DNA synthesis by inhibiting cell progression from the G1 to S phase, thereby suppressing cancer cell proliferation.^12^ In Japan, oral palbociclib and abemaciclib are newly approved for hormone receptor-positive and HER2-negative mBC treatment. However, ribociclib has not been approved; a reason for this is the high toxicity profile, including abnormal hepatic function in the Japanese population.^13^

The concomitant use of proton pump inhibitors (PPIs) substantially decreases PFS in patients with mBC treated with palbociclib and ribociclib.^14-16^ The potential mechanism of these drug-drug interactions (DDIs) may include gastric pH elevation by PPIs. PPIs reduce the bioavailability of several anticancer drugs through dissolution and absorption.^17-20^ Conversely, several studies do not support the existence of the DDIs.^21-24^ Therefore, the impact of co-administration of PPIs with CDK4/6 inhibitors remains controversial. These previous studies shared a retrospective, observational design and included patients with hormone receptor-positive and HER2-negative mBC. However, in several of those studies, treatment lines and the corresponding patient eligibility criteria were not specified and the sensitivity to endocrine therapy of the patients were not considered. The PFS of endocrine-sensitive patients receiving palbociclib was reportedly 24.8 months, whereas that in endocrine-resistant patients was only 9.5 months.^4,5^ As these different results cannot be equally evaluated, in the present study, we pursued in patients with endocrine resistance who had never undergone chemotherapy, because we thought that the overall survival (OS) of endocrine-sensitive patients treated with CDK4/6 inhibitors is influenced by second-line or subsequent therapy and that a 5-year follow-up may be too short since endocrine-sensitive patients have a relatively favorable prognosis. To the best of our knowledge, the potential DDIs mentioned above have not yet been investigated in the Japanese population, and no studies are available on the DDIs between PPIs and abemaciclib. Thus, we hypothesized that concomitant PPI use decreases the effectiveness of CDK4/6 inhibitors. The present study aimed to clarify, using real-world data, whether concomitant PPI use alters the effectiveness of palbociclib and abemaciclib in patients with hormone receptor-positive and HER2-negative mBC.

## Patients and Methods

### Study Design

This multicenter, retrospective, observational, case-control study was conducted across 4 medical institutions: National Cancer Center Hospital East (Chiba, Japan), Keio University Hospital (Tokyo, Japan), Miyagi Cancer Center (Miyagi, Japan), and Gifu University Hospital (Gifu, Japan). Patient data were obtained from the medical records of each institution. Data integration and subsequent analyses were performed at the Keio University Faculty of Pharmacy (Tokyo, Japan). The methodology adopted herein adhered to the Strengthening the Reporting of Observational Studies in Epidemiology statement.^25^

The eligibility criteria for the patients were as follows: (1) aged ≥ 18 years and consecutively presenting with a diagnosis of hormone receptor-positive and HER2-negative mBC (hormone receptor-positive was defined as tumors with estrogen and/or progesterone receptor expression > 1%; HER2-negative was defined as a score of 0, 1+, or 2 + using immunohistochemistry; and negative result as per in situ hybridization^11^); (2) received palbociclib (125 mg taken orally once daily for 21 consecutive days followed by 7 days off in 28-day cycles) or abemaciclib (150 mg taken orally twice daily) plus endocrine therapy for the first time between December 2017 and August 2022; (3) underwent second-line or subsequent-line endocrine therapy (for patients who have de novo metastatic cancer or experienced progression during neoadjuvant chemotherapy prior to surgery); (4) underwent first-line or subsequent-line endocrine therapy (for patients who experienced progression during treatment or within 12 months of completion of adjuvant endocrine therapy, and (5) underwent second-line or subsequent-line endocrine therapy (for patients who developed recurrent disease > 12 months of completing that treatment). These definitions of first- and second-line endocrine therapies were based on the American Society of Clinical Oncology guidelines 2016.^26^ The dose reduction of CDK4/6 inhibitors and clinical follow-up were modified at the clinician’s discretion according to the efficacy and/or safety profile of each patient.

The exclusion criteria were as follows: (1) refusal to use medical records for research; (2) insufficient or missing data; and (3) history of chemotherapy for disease control of mBC prior to treatment with CDK4/6 inhibitors, except for neoadjuvant or adjuvant chemotherapy.

### Data Collection

Data of patients were de-identified and managed anonymously. Collected data included age, sex, menopausal status, Eastern Cooperative Oncology Group performance status (ECOG PS), metastatic site, number of metastatic sites, medical history (including CDK4/6 inhibitor and PPI use), treatment line, date of progression and/or death at the time of initiation of CDK4/6 inhibitors, and the incidence of adverse events of grade ≥ 3 while taking CDK4/6 inhibitors or up to 3 months after taking. The date of progression was defined as the first incidence of disease progression based on computed tomography using the Response Evaluation Criteria in Solid Tumours criteria version 1.1^27^ or clinical progression. Adverse events were graded according to the Common Terminology Criteria for Adverse Events version 5.0.^28^ Each attending physician advised against the intake of strong inhibitors or inducers of cytochrome P450 3A4 based on their knowledge. Treatment groups were defined as “concomitant use of PPIs” if PPI administration covered the entire or more than half of the treatment period with palbociclib^15,24^ or “no concomitant use of PPIs” if PPI administration covered less than half of the treatment period. Moreover, “visceral” refers to lung, liver, brain, pleural, and peritoneal involvement. The follow-up period ended on November 30, 2022.

### Endpoints

The efficacy endpoints were PFS and OS when treated with or without PPIs. The safety endpoint was adverse events when treated with or without PPIs. PFS was defined as the period from the date of initiation of CDK4/6 inhibitors to the date of disease progression or death from any cause, whereas OS was defined as the period from the date of initiation of CDK4/6 inhibitors to the date of death from any cause. Patients without documented progressive disease or those who were still alive were censored for PFS and OS, respectively, on the date of the last follow-up.

### Statistical Analysis

Baseline patient characteristics are reported as median (interquartile range) for continuous variables and proportions for categorical variables. PFS and OS were estimated using the Kaplan-Meier method and compared using a log-rank test. Propensity score-matched analysis was performed to minimize potential selection bias due to lack of randomization.^29^ Propensity scores of concomitant PPI use were estimated using a logistic regression model based on the following clinically selected covariates: age, ECOG PS (0-1 *vs.* 2), disease site (visceral *vs*. non-visceral), number of metastases, and previous lines of endocrine treatment (1 *vs*. ≥ 2). In a sensitivity analysis, two propensity score-adjusted analyses were performed: (1) a multivariable Cox proportional hazards model including the propensity score of concomitant use of PPIs as a covariate and (2) the inverse probability of treatment weighting (IPTW) method.^30^ The results are presented as hazard ratios (HR) and 95% CIs. As a post hoc analysis, the Bayesian posterior probability of HRs from 0.83 to 1.2 based on non-informative prior distribution was computed to assess the equivalence.^31^ The follow-up time was calculated using the reverse Kaplan-Meier estimate.^32^ All statistical analyses were performed using SAS version 9.4 (SAS Institute Inc., Cary, NC, USA) and SPSS Statistics version 29 (IBM, Armonk, NY, USA). All *P*-values were 2 sided, and the significance level was set at 0.05.

### Ethics Statement

The study protocol was representatively approved by the Ethics Committees of the Keio University School of Medicine (approval number: 20221136). Permission to conduct the present study was then obtained from each facility. This study was conducted in accordance with the principles of the Declaration of Helsinki and the Ethical Guidelines for Medical and Health Research involving Human Subjects by the Ministry of Education, Culture, Sports, Science, and Technology and the Ministry of Health, Labour, and Welfare of Japan. The requirement for written informed consent was waived by the ethics review committees owing to the retrospective nature of this study. Accordingly, we provided patients with an opt-out method through the official website of each hospital.

## Results

### Patient Characteristics

**Figure 1.**
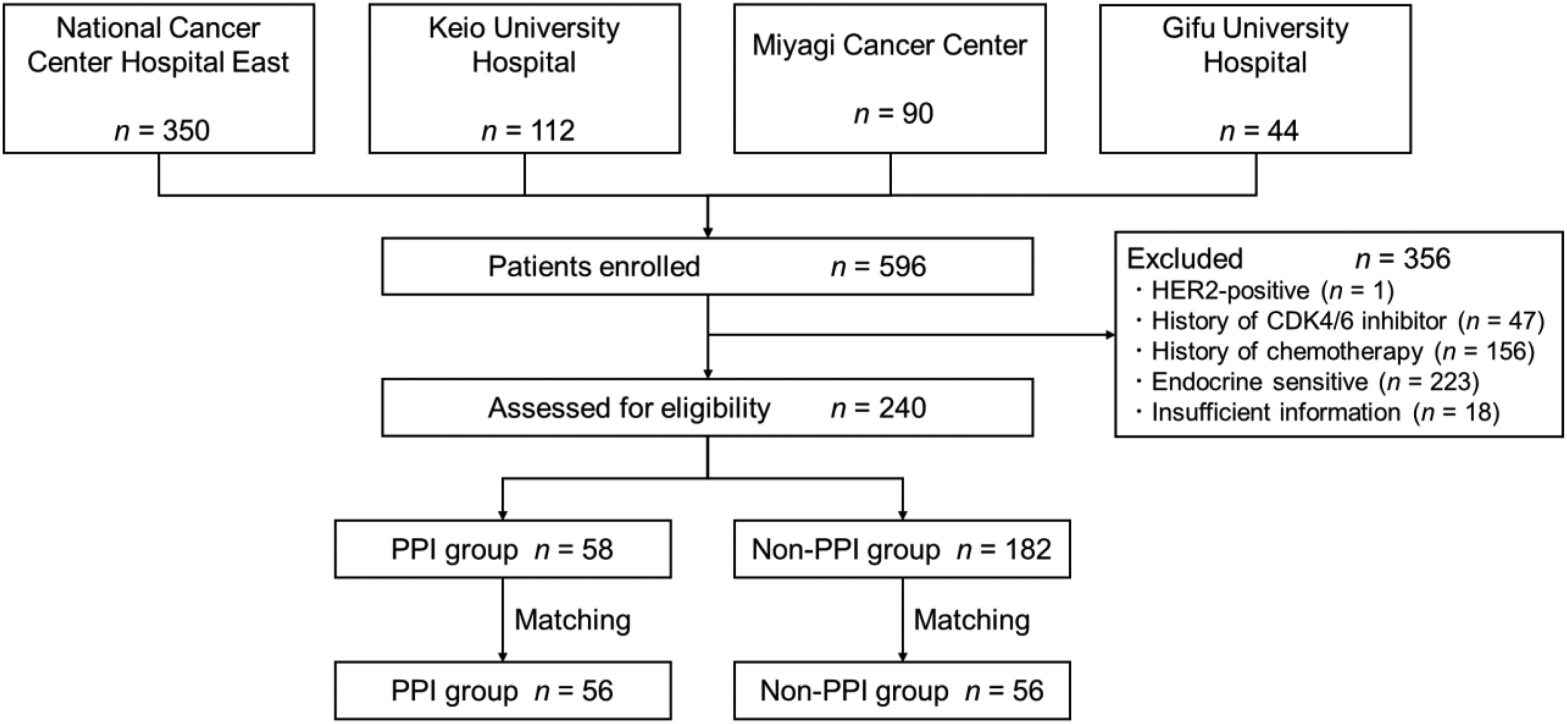
Patient enrollment flowchart. Abbreviations: HER2, human epidermal growth factor receptor type 2; CDK4/6, cyclin-dependent kinases 4 and 6; PPI, proton pump inhibitor.

Figure 1 shows the patient enrollment flowchart. Overall, 596 patients were initially surveyed at the 4 sites, and 240 met the eligibility criteria. Among them, 58 patients were taking CDK4/6 inhibitors with PPIs. Propensity score-matched analysis resulted in 112 patients, who were divided into groups treated with (PPI group; *n* = 56) and without (non-PPI group; *n* = 56) PPIs. After matching, the distributions of the propensity score in both groups were similar (Supplementary Fig. S1). Table 1 presents the baseline patient characteristics. The median age of the patients was 71 (interquartile range, 61-75) years. Of the 78 patients who were receiving palbociclib, 58 and 20 were taking it as capsules and tablets, respectively. Approximately 70% of patients used CDK4/6 inhibitors in the first- or second-line endocrine therapy. The number of metastases was within 5, and more than half of the patients had visceral metastases. Approximately two-thirds of the patients in both groups required dose reduction of CDK4/6 inhibitors, and no significant difference in dosage between the two groups was noted. In total, 66% of the concomitant endocrine therapy included fulvestrant use. Lansoprazole was the most commonly prescribed PPI (45%).

**Table 1.** Patient characteristics.

Characteristics	Unmatched patients (*n *= 240)		Matched patients (*n *= 112)	
	PPI (*n *= 58)	Non-PPI (*n *= 182)	PPI (*n *= 56)	Non-PPI (*n *= 56)
Age (years), median (IQR)^a^	71 (60-75)	62 (50-70)	71 (60-75)	70 (62-75)
Sex				
Female	57 (98)	180 (99)	55 (98)	55 (98)
Male	1 (2)	2 (1)	1 (2)	1 (2)
Menopause				
Premenopause	8 (14)	47 (26)	8 (14)	4 (7)
Postmenopause (or male sex)	50 (86)	135 (74)	48 (86)	52 (93)
ECOG PS^a^				
0-1	52 (90)	178 (98)	52 (93)	53 (95)
2	6 (10)	4 (2)	4 (7)	3 (5)
Treatment line^a^				
1st	16 (28)	70 (39)	16 (29)	15 (27)
2nd	25 (43)	70 (39)	23 (41)	26 (46)
≥ 3rd	17 (29)	42 (23)	17 (30)	15 (27)
Number of metastases, median (IQR)^a^	2 (1-3)	2 (1-3)	2 (1-3)	2 (1-3)
Metastatic site^a^				
Visceral	36 (62)	107 (59)	34 (61)	36 (64)
Non-visceral	22 (38)	75 (41)	22 (39)	20 (36)
CDK4/6 inhibitor				
Palbociclib capsule	34 (59)	91 (50)	33 (59)	25 (45)
Palbociclib tablet	11 (19)	41 (23)	10 (18)	10 (18)
Abemaciclib	13 (22)	50 (28)	13 (23)	21 (38)
Dose reduction of CDK4/6 inhibitor				
Yes	36 (62)	125 (69)	36 (64)	40 (71)
No	22 (38)	57 (31)	20 (36)	16 (29)
Concomitant endocrine therapy				
Anastrozole	2 (3)	19 (10)	2 (4)	7 (13)
Letrozole	15 (26)	34 (19)	15 (27)	11 (20)
Exemestane	1 (2)	4 (2)	0	2 (4)
Tamoxifen	0	1 (1)	0	1 (2)
Fulvestrant	40 (69)	124 (68)	39 (70)	35 (63)
Concomitant LH–RH agonist				
None	51 (88)	139 (76)	49 (88)	53 (95)
Leuprorelin acetate	5 (9)	35 (19)	5 (9)	3 (5)
Goserelin acetate	2 (3)	8 (4)	2 (4)	0
PPI used				
Omeprazole	3 (5)		3 (5)	
Lansoprazole	27 (47)		25 (45)	
Rabeprazole	8 (14)		8 (14)	
Esomeprazole	10 (17)		10 (18)	
Vonoprazan	10 (17)		10 (18)	

Abbreviations: IQR, interquartile range; PPI, proton pump inhibitor; ECOG PS, Eastern Cooperative Oncology Group performance status; CDK4/6 inhibitor, cyclin-dependent kinases 4 and 6; LH–RH, luteinizing hormone-releasing hormone.

Data are presented as the number (%) of patients unless otherwise indicated. Percentages have been rounded and may not total 100.

^a^Age, ECOG PS, treatment line, number of metastases, and metastatic sites were used to match the two groups.

### Efficacy

**Figure 2.**
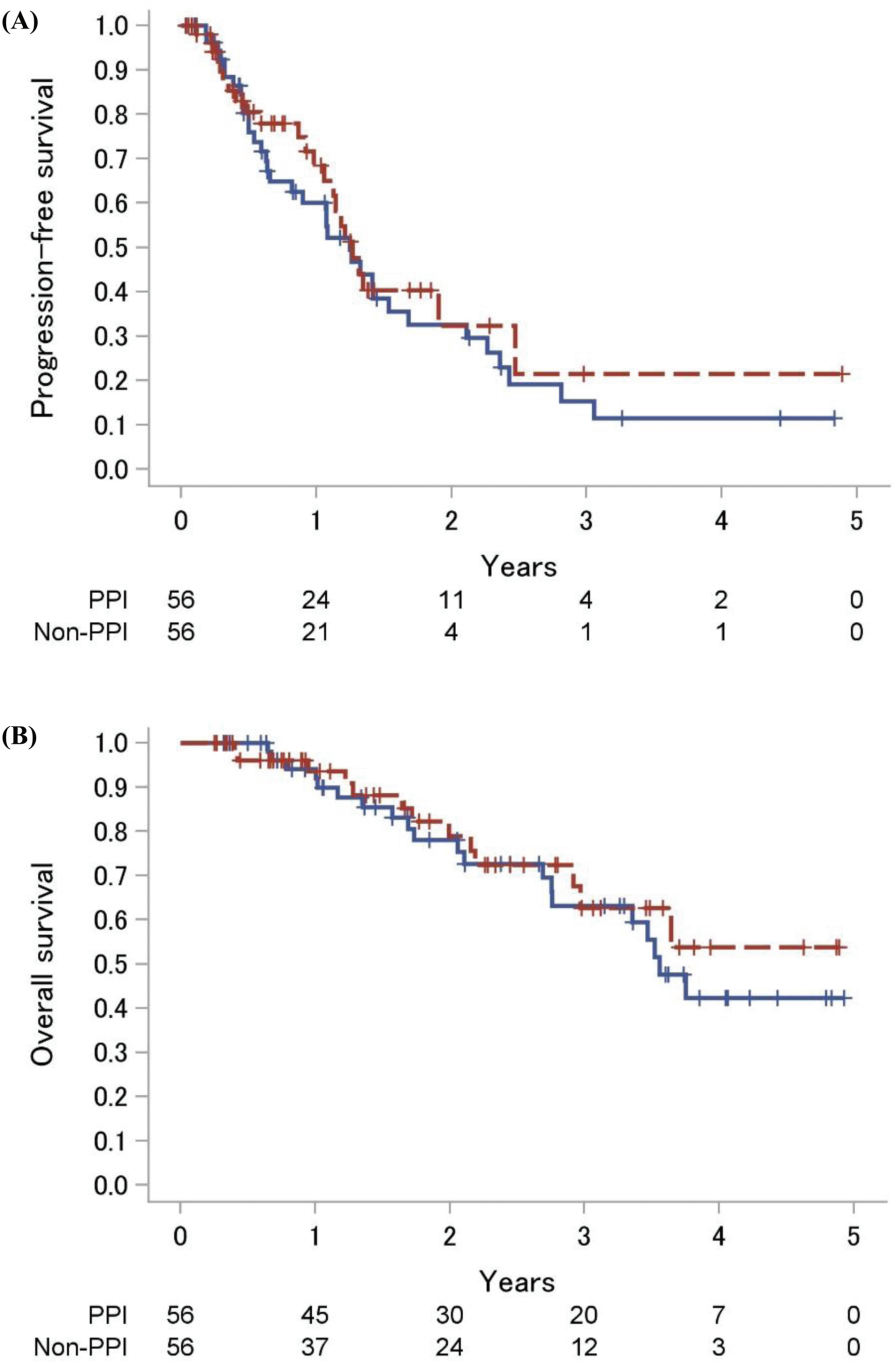
Kaplan-Meier survival curves according to concomitant or non-concomitant PPI use. Kaplan-Meier survival curves were constructed according to the use of PPIs in the propensity score-matched patients. Solid and dashed lines represent the PPI and non-PPI groups, respectively. The number of patients at risk are shown at the bottom. (A) Progression-free survival. (B) Overall survival. Abbreviation: PPI, proton pump inhibitor.

The median follow-up time was 2.6 (95% CI, 2.2-3.1) years. The median PFS was 1.2 (95% CI, 0.7-1.7) and 1.3 (95% CI, 1.1-2.5) years in the PPI and non-PPI groups, respectively (log-rank test: *P* = 0.53; Fig. 2). The crude HR was 1.19 (95% CI, 0.70-2.02) according to the univariable analysis. The median OS period was 3.6 (95% CI, 2.8-not reached) years in the PPI group, whereas it was not reached in the non-PPI group (log-rank test: *P* = 0.57). The crude HR was 1.23 (95% CI, 0.61-2.47) according to the univariable analysis.

Sensitivity analyses using a propensity score-adjusted model and IPTW analysis for PFS revealed consistent results (HR, 1.11; 95% CI, 0.73-1.68; *P *= 0.63 and HR, 1.09; 95% CI, 0.76-1.57; *P *= 0.62, respectively). Similar results were obtained for OS using propensity score-adjusted and IPTW analyses (HR, 1.21; 95% CI, 0.68-2.15; *P *= 0.51 and HR, 1.26; 95% CI, 0.71-2.24; *P *= 0.43, respectively). The Bayesian posterior probability of HRs for the PFS from 0.83 to 1.2 ranged from 42% to 77% (Table 2).

**Table 2.** HRs of PFS, OS, and the Bayesian posterior probability of HRs from 0.83 to 1.2.

	HR (95% CI)	*P*-value	Posterior probability (%)
PFS			
Propensity score matching	1.19 (0.70-2.02)	0.53	42
Multivariable analysis	1.11 (0.73-1.68)	0.63	57
IPTW	1.09 (0.76-1.57)	0.62	77
OS			
Propensity score matching	1.23 (0.61-2.47)	0.57	34
Multivariable analysis	1.21 (0.68-2.15)	0.51	39
IPTW	1.26 (0.71-2.24)	0.43	38

Abbreviations: HR, hazard ratio; CI, confidence interval; PFS, progression-free survival; OS, overall survival; IPTW, inverse probability of treatment weighting.

Subgroup analyses were performed separately for palbociclib (*n* = 177) and abemaciclib (*n* = 63) without propensity score matching. The median PFS in the palbociclib group was 1.3 (95% CI, 0.6-2.3) and 1.1 (95% CI, 0.9-1.3) years in the PPI and non-PPI groups, respectively (Supplementary Fig. S2), with no significant difference (HR, 0.94; 95% CI, 0.61-1.46; *P *= 0.80). Similarly, OS did not differ significantly (HR, 1.47; 95% CI, 0.82-2.62; *P *= 0.19).

Similar results were observed for abemaciclib (PFS: HR, 1.30; 95% CI, 0.53-3.17; *P *= 0.56 and OS: HR, 1.22; 95% CI, 0.33-4.47; *P *= 0.76) (Supplementary Fig. S3).

Additionally, 125 and 52 patients were classified into the palbociclib capsule and tablet groups, respectively. The analysis indicated no significant differences in either PFS or OS for the capsule (PFS: HR, 1.06; 95% CI, 0.66-1.71; *P *= 0.80 and OS: HR, 1.40; 95% CI, 0.76-2.58; *P *= 0.28) (Supplementary Fig. S4). Similar results were obtained for the tablets (data not shown).

### Safety

Table 3 presents grade 3/4 adverse events in the matched population. The proportion of adverse events was similar for each CDK4/6 inhibitor, regardless of PPI use. Palbociclib was associated with a high incidence of neutrophil count decreased, whereas abemaciclib was associated with a high incidence of diarrhea. The PPI and non-PPI groups did not differ significantly (Fisher’s exact tests: *P* = 0.75 and *P* = 1.00, respectively). No grade 5 adverse events were observed.

**Table 3.** Grade 3/4 adverse events.

	Palbociclib (*n* = 78)		Abemaciclib (*n* = 34)	
	PPI (*n* = 43)	Non-PPI (*n* = 35)	PPI (*n* = 13)	Non-PPI (*n* = 21)
Hematological toxicity				
Neutrophil count decreased	36 (84)	31 (89)	7 (54)	4 (19)
White blood cell count decreased	20 (47)	11 (31)	2 (15)	0
Anemia	5 (11)	3 (9)	1 (8)	3 (14)
Platelet count decreased	4 (9)	1 (3)	0	0
Febrile neutropenia	0	0	1 (8)	0
Non-hematological toxicity				
Diarrhea	1 (2)	0	3 (23)	4 (19)
Pneumonitis	3 (7)	2 (6)	0	2 (10)
AST level increased	0	2 (6)	1 (8)	2 (10)
ALT level increased	0	1 (3)	1 (8)	2 (10)
Stevens–Johnson syndrome	0	0	1 (8)	0
Skin ulceration	1 (2)	0	0	0
Malaise	0	1 (3)	0	0
Skin infection	0	1 (3)	0	0
Appendicitis	0	1 (3)	0	0
Nausea	0	0	0	1 (5)
Anorexia	0	0	1 (8)	0

Abbreviations: PPI, proton pump inhibitor; AST, aspartate aminotransferase; ALT, alanine aminotransferase.

Data are presented as the number (%) of patients.

## Discussion

No study to date has focused on the association between concomitant PPI use and the effectiveness of CDK4/6 inhibitors in patients with endocrine resistance who have never undergone chemotherapy. In the present study, we found that concomitant PPI use did not significantly reduce PFS and OS in propensity score-matched patients. Additionally, the sensitivity and subgroup analyses showed consistent results. To the best of our knowledge, this is the first multicenter study examining the effect of PPIs co-administered with CDK4/6 inhibitors on survival outcomes in Japanese patients with endocrine resistance in a real-world setting.

The study population was relatively homogeneous, with a large number of survival events (i.e., PFS and OS). We focused on patients resistant to endocrine therapy who had never undergone chemotherapy. This was determined with reference to eligible patients in the PALOMA-3 phase III trial.^5^ We considered this population suitable for clarifying whether concomitant PPI use alters the effectiveness of palbociclib and abemaciclib; the efficacy did not differ significantly between the groups treated with and without PPIs in the sensitivity and subgroup analyses. These results did not support our hypothesis that concomitant PPI use decreases the effectiveness of CDK4/6 inhibitors. Sun et al^33^ stated the pharmacokinetics of palbociclib capsules with PPIs. Under fasting conditions, co-administration of PPIs and palbociclib capsules decreased the palbociclib mean area under the concentration-time curve from time 0 to infinity by 62%; however, under fed conditions, it decreased by only 13%. If patients take palbociclib capsules appropriately after meals, the effect of PPIs on palbociclib blood levels may not be clinically significant. The differences between these DDI studies may be attributed to differences in the healthcare systems for outpatients among countries. The present study was conducted at university hospitals with more than 600 beds and designated cancer centers in Japan, wherein patients are managed by high-level experts in cancer treatment. Particularly, at the National Cancer Center Hospital East, patients received individual guidance from hospital pharmacists regarding oral anticancer drugs. Patient education is essential for developing adherence. In Japan, hospital pharmacists provide patients with adequate education and follow-up on medications.^34^ Moreover, the most important feature of the Japanese healthcare system is the separation of prescription and dispensary; community pharmacies provide thoughtful guidance to outpatients regarding oral anticancer drugs.^35^ Therefore, we consider that our patients sufficiently well-educated to take the palbociclib capsule after meals according to the package insert of the capsule. We believe that these factors are associated with the effectiveness of CDK4/6 inhibitors in patients treated with PPIs. This hypothesis is supported by the finding that dose reduction of CDK4/6 inhibitors was independent of concomitant PPI use. Assuming PPIs reduce the blood concentration of CDK4/6 inhibitors, more patients in the non-PPI group than in the PPI group should have required dose reduction of CDK4/6 inhibitors. Notably, the safety was similar between the two groups. We posited that if concomitant PPI use was to reduce the blood concentration of CDK4/6 inhibitors, the incidence and severity of adverse events should decrease; however, this trend was not observed. This result can be explained in the same manner as the efficacy results.

To date, no pharmacokinetic data on DDIs between abemaciclib and PPIs have been reported. The abemaciclib tablet formulation is weakly basic, and its solubility is expected to be pH-dependent. Abemaciclib has structural characteristics similar to those of palbociclib, potentially explaining the consistent results. To the best of our knowledge, this is the first study to demonstrate the DDIs between abemaciclib and PPIs. Abemaciclib is approved for mBC, and as an adjuvant therapy, it can be used in more patients than palbociclib,^8,9,36^ highlighting the potential clinical significance of our findings.

Concomitant PPI use reportedly reduces the effectiveness of CDK4/6 inhibitors.^14-16^ Our study is consistent with these previous reports in sharing a retrospective, observational design for patients with hormone receptor-positive and HER2-negative mBC. However, there are some contrasting findings.^21-24^ In contrast to our results, Lee et al^16^ reported DDIs between palbociclib capsules and PPIs in 1,310 patients and concluded, using propensity score matching, that concomitant PPI use significantly reduced the effectiveness of palbociclib capsules. The authors attributed this result to the decreased solubility of palbociclib capsules. However, ECOG PS and exact PFS were not evaluated because they used insurance claims data. Therefore, the time to the next treatment was alternatively used for PFS. However, this evaluation method is inaccurate. In the present study, both palbociclib capsules (to be taken after meals) and tablets (can be taken regardless of meals) were included, with no reduced efficacy observed. Given the reformulation of palbociclib from capsules to tablets, DDIs between palbociclib tablets and PPIs are unlikely to remain clinically relevant.

This study has various strengths. First, it included patients from 4 medical institutions across Japan. Therefore, our data may be applicable to similar populations in clinical settings and will presumably play a crucial role in shared decision-making between patients and a multidisciplinary care team comprising doctors, pharmacists, and nurses. In particular, we believe that this study provides valuable evidence for their decision to first-line endocrine therapy for patients who experienced progression during treatment or within 12 months of completion of adjuvant endocrine therapy. Second, we focused on patients with endocrine-resistant mBC who had never undergone chemotherapy. Third, the follow-up period was relatively long. We evaluated both the PFS and OS. Finally, we performed propensity score matching, resulting in both groups being well-balanced. This analysis increases the robustness of the results and is a novel feature of our study.

### Limitations

Our study has some limitations. First, this was a retrospective, observational, case-control study; therefore, information bias exists. Second, the cohort size was small; therefore, we could not include any additional factors for propensity score matching and were unable to match the types of CDK4/6 inhibitors. Third, no exact data on adherence to CDK4/6 inhibitors or PPIs were obtained; we used prescription history. Additionally, we were unable to perform quantitative or qualitative assessments to establish the patterns of drug timing. Fourth, this study was a discovery cohort. Therefore, our findings need to be validated in future studies. Fifth, no pharmacokinetic data for the CDK4/6 inhibitors were obtained. Therefore, prospective pharmacokinetic studies should be conducted. Finally, the current standard treatment is the use of CDK4/6 inhibitors and endocrine therapy drugs as first-line treatments for patients with hormone receptor-positive and HER2-negative mBC. Therefore, studies in patients with endocrine sensitivity would be more applicable to current practice than studies in endocrine-resistant populations. We aim to evaluate the outcomes for patients in the first-line setting treated with CDK4/6 inhibitors and endocrine therapy drugs in future investigations.

## Conclusions

This study suggests that the effectiveness of CDK4/6 inhibitors is unlikely to be affected by concomitant PPI use in Japanese patients with hormone receptor-positive and HER2-negative endocrine-resistant mBC. Further consideration should be given to changing the prescription of PPIs for patients taking palbociclib or abemaciclib. Our findings may also be generalizable to Japanese patients, warranting future prospective pharmacokinetic studies.

## Supplementary Material

oyae015_suppl_Supplementary_Figures_1-4

## Data Availability

The data underlying this article cannot be shared publicly due to the privacy of individuals that participated in the study. The data will be shared on reasonable request to the corresponding author.
