# Supplementary material for: Proton Pump Inhibitors and Cyclin-Dependent Kinase 4/6 Inhibitors in Patients With Breast Cancer
Source: Oncologist. 2024 Feb 10;29(6):e741–9. doi: 10.1093/oncolo/oyae015 (PMC11144975; doi:10.1093/oncolo/oyae015)
Supplement: oyae015_suppl_Supplementary_Figures_1-4 [file oyae015_suppl_supplementary_figures_1-4.docx]

**Supplementary Figures**

**
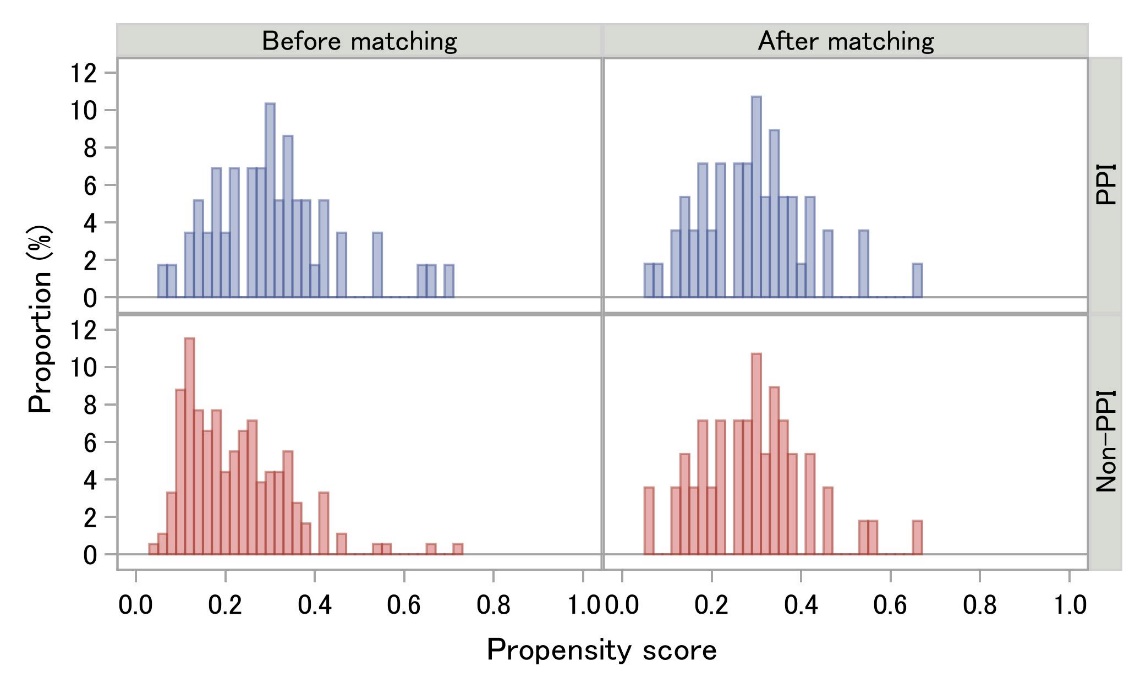
**

**Supplementary Figure 1.** Histograms of propensity score before and after matching

**(A)**

**
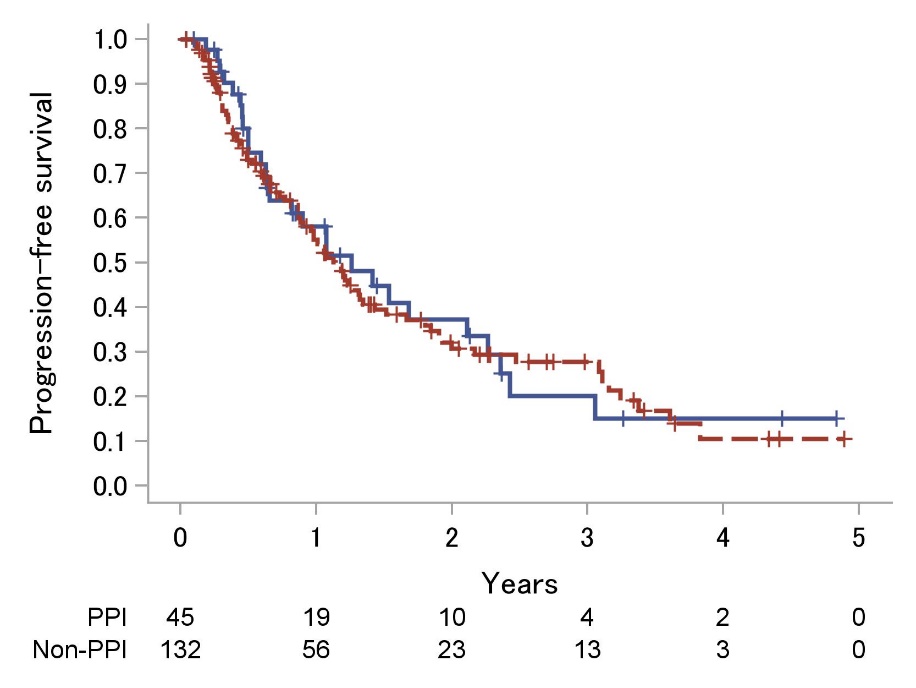
**

**(B)**

**
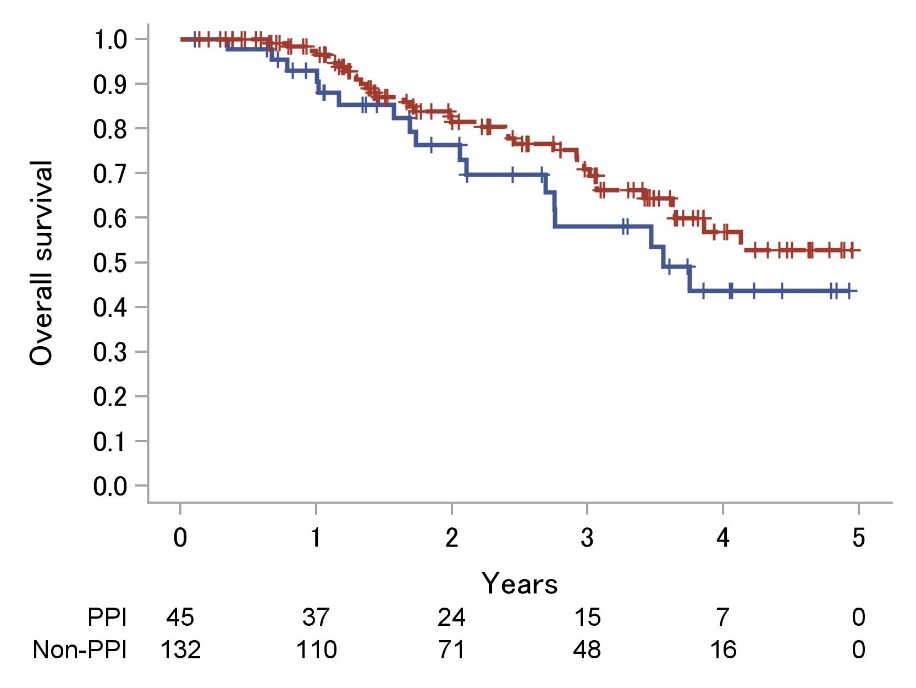
**

**Supplementary Figure 2.** Kaplan–Meier survival curves according to concomitant or non-concomitant PPI use in the palbociclib group. Solid and dashed lines represent the PPI and non-PPI groups, respectively. The numbers at risk are shown at the bottom. (A) Progression-free survival. (B) Overall survival. Abbreviation: PPI, proton pump inhibitor

**(A)**

**
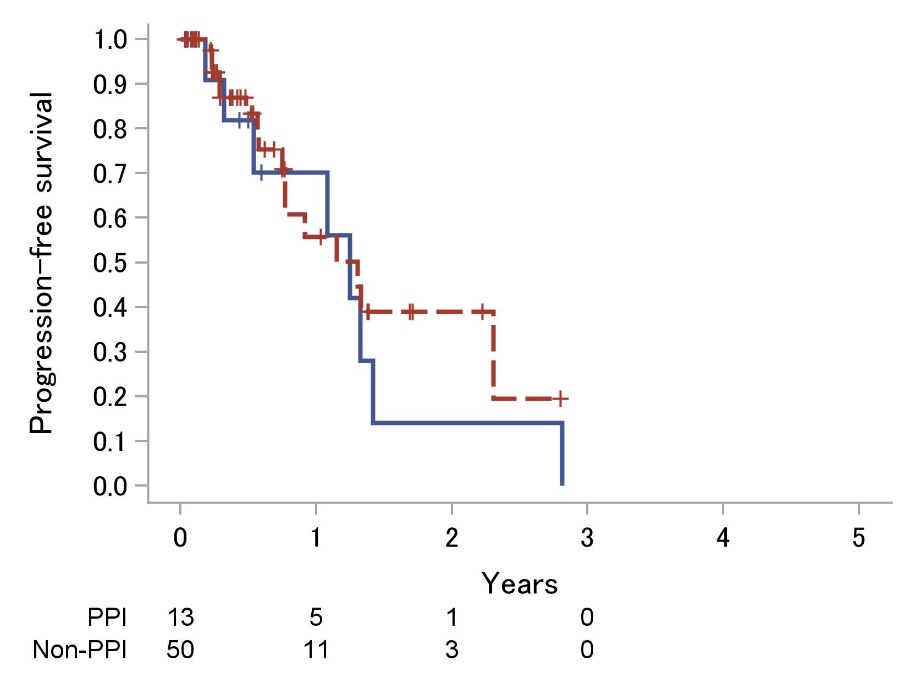
**

**(B)**

**
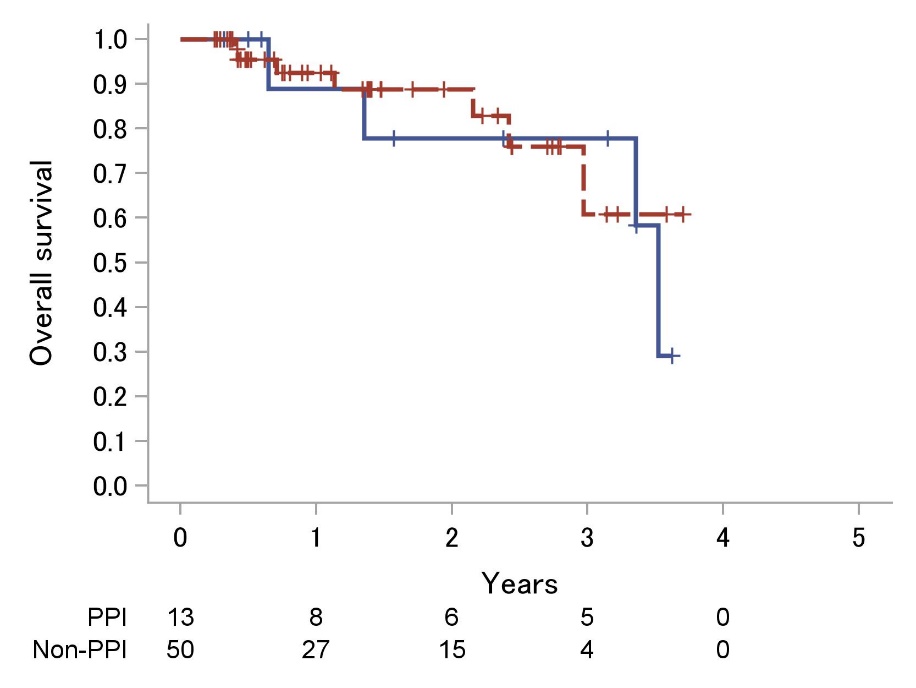
**

**Supplementary Figure 3.** Kaplan–Meier survival curves according to concomitant or non-concomitant PPI use in the abemaciclib group. Solid and dashed lines represent the PPI and non-PPI groups, respectively. The numbers at risk are shown at the bottom. (A) Progression-free survival. (B) Overall survival. Abbreviation: PPI, proton pump inhibitor

**(A)**

**
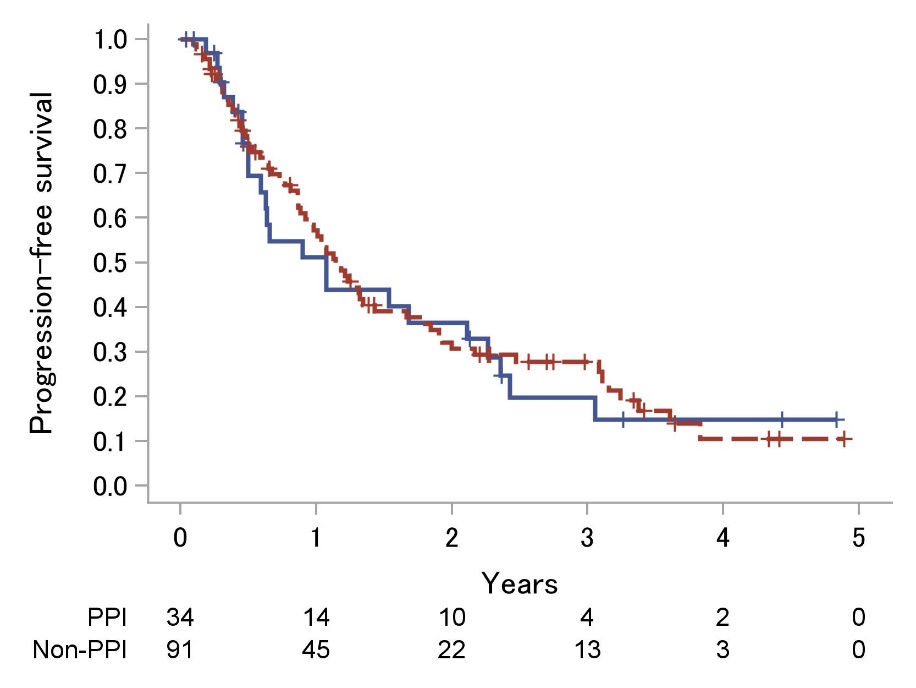
**

**(B)**

**
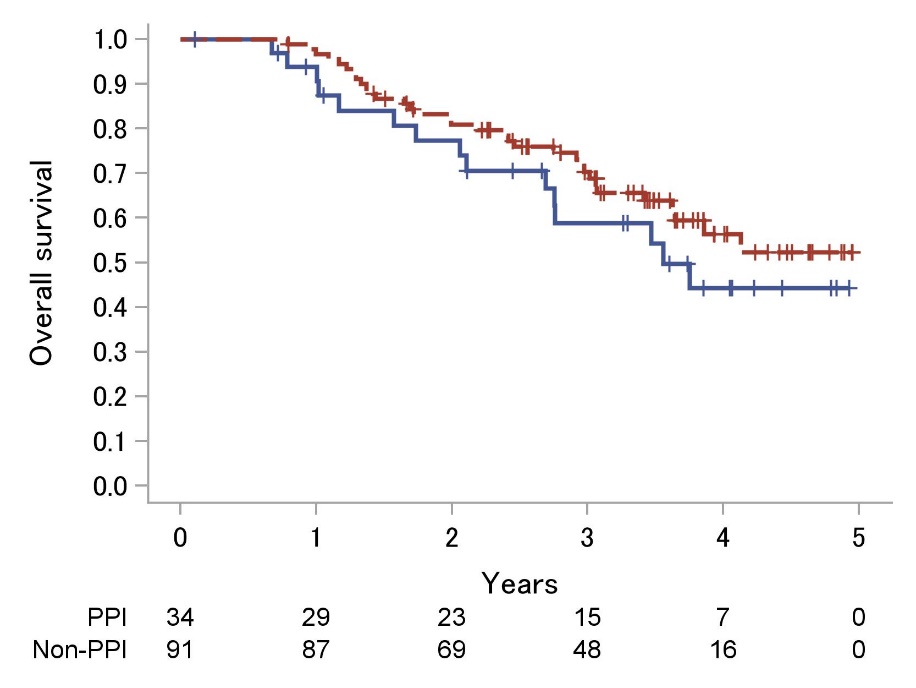
**

**Supplementary Figure 4.** Kaplan–Meier survival curves according to concomitant or non-concomitant PPI use in the palbociclib capsules group. Solid and dashed lines represent the PPI and non-PPI groups, respectively. The numbers at risk are shown at the bottom. (A) Progression-free survival. (B) Overall survival. Abbreviation: PPI, proton pump inhibitor
